# Engineering a Novel Modular Adenoviral mRNA Delivery Platform Based on Tag/Catcher Bioconjugation

**DOI:** 10.3390/v15112277

**Published:** 2023-11-20

**Authors:** Kexin Geng, Paul J. Rice-Boucher, Elena A. Kashentseva, Igor P. Dmitriev, Zhi Hong Lu, S. Peter Goedegebuure, William E. Gillanders, David T. Curiel

**Affiliations:** 1 Department of Radiation Oncology, Biologic Therapeutics Center, Washington University School of Medicine, St. Louis, MO 63110, USA; g.kexin@wustl.edu (K.G.); pboucher@wustl.edu (P.J.R.-B.); ekashentseva@wustl.edu (E.A.K.); idmitriev@wustl.edu (I.P.D.); zhihonglu@wustl.edu (Z.H.L.); 2 Department of Biomedical Engineering, McKelvey School of Engineering, Washington University in Saint Louis, St. Louis, MO 63130-4899, USA; 3 Department of Surgery, Washington University School of Medicine, St. Louis, MO 63110, USA; goedegep@wustl.edu (S.P.G.); gillandersw@wustl.edu (W.E.G.); 4 Alvin J. Siteman Cancer Center at Barnes-Jewish Hospital and Washington University School of Medicine, St. Louis, MO 63110, USA

**Keywords:** mRNA delivery, adenovirus, Catcher/Tag bioconjugation, RNA binding protein, mRNA condensation, vaccine

## Abstract

mRNA vaccines have attracted widespread research attention with clear advantages in terms of molecular flexibility, rapid development, and potential for personalization. However, current mRNA vaccine platforms have not been optimized for induction of CD4/CD8 T cell responses. In addition, the mucosal administration of mRNA based on lipid nanoparticle technology faces challenges in clinical translation. In contrast, adenovirus-based vaccines induce strong T cell responses and have been approved for intranasal delivery. To leverage the inherent strengths of both the mRNA and adenovirus platforms, we developed a novel modular adenoviral mRNA delivery platform based on Tag/Catcher bioconjugation. Specifically, we engineered adenoviral vectors integrating Tag/Catcher proteins at specific locales on the Ad capsid proteins, allowing us to anchor mRNA to the surface of engineered Ad viruses. In proof-of-concept studies, the Ad-mRNA platform successfully mediated mRNA delivery and could be optimized via the highly flexible modular design of both the Ad-mRNA and protein bioconjugation systems.

## 1. Introduction

mRNA-based therapeutics have unlocked a new era for disease prevention and treatment. The delivery of mRNAs encoding pathogen or cancer antigens, gene-editing components, and therapeutic proteins has enabled the development of new medicines, including applications in gene therapy, immunotherapy, gene-editing, and vaccines. As the intermediate molecule between DNA and protein, mRNA-based platforms have several advantages over conventional DNA-based approaches, including negligible risk of insertional mutagenesis, a minimal requirement for cytoplasmic rather than nuclear delivery, and one-step translation [1,2,3]. In addition, proteins expressed from delivered mRNAs generally possess relatively longer-lasting effects and better durability than directly administered proteins [4]. The remarkably rapid development and clinical approval of two mRNA-based COVID vaccines, Moderna mRNA-1273 and Pfizer/BioNTech BNT162b2, highlight the capacity of mRNA for quick therapeutic development in response to acute disease when supported by well-established manufacturing processes. The application of mRNA technology has thus attracted widespread research attention across various fields, fueling the revolution in mRNA engineering and delivery. 

Pioneering studies of mRNA as a vaccine date back to the early 1990s, when several groups demonstrated the ability of mRNA to induce an immune response in mice by injecting mRNA [5]. However, further investigations and clinical translation were hindered by the inherent limitations of mRNA, including its instability, immunogenicity, and poor translation. To address these challenges, researchers have undertaken intensive studies in mRNA engineering, including optimization of the untranslated region, the addition of a poly(A) tail, incorporation of pseudouridine, and the development of novel mRNA constructs, such as circular RNA and self-amplifying RNA [6,7,8,9,10,11]. These advances have allowed for the successful clinical translation of a range of mRNA therapies. 

The development of mRNA delivery vehicles, including lipid nanoparticles (LNPs), polymers, and peptides, has also facilitated mRNA expression and clinical translation. Among these, LNPs have achieved the greatest clinical success and have been leveraged in both approved mRNA-based COVID vaccines [12,13]. Despite these successes, current LNP formulations could be improved in several important ways. Notably, mRNA vaccines primarily induce a humoral B cell response [14]. However, some pathogens or diseases require successful induction of CD4/CD8 T cell responses. This could be achieved through targeting defined cell types, such as dendritic cells (DCs) which are pivotal in activating and mediating the T cell response [15,16]. This pathway is particularly critical in cancer, and selected mRNA cancer vaccines in development incorporate DC-targeting strategies to facilitate CD4/CD8 T cell responses [17]. In addition, delivery of antigens via the intranasal route is an effective approach to induce potentially sterilizing immunity in the upper and lower respiratory tracts, which act as a first-line barrier against the transmission of respiratory pathogens [18]. This route is therefore an attractive option for limiting the spread of respiratory diseases such as COVID-19 and has the potential to be pivotal in controlling future pandemics. Although a few studies have explored the use of LNPs to deliver mRNA intranasally, achieving success in clinical trials seems to be challenging [19]. 

In contrast, adenoviral vectors (Ads) induce strong cellular immune responses, and some formulations have been approved for intranasal delivery [20]. Ads are icosahedral non-enveloped DNA viruses, generally responsible for the common cold, that were first discovered and isolated in 1953 [21,22]. Since then, it has become one of the most widely used tools for gene therapy and vaccine development due to its well-deciphered viral genome and intensively studied biological structure. This strong scientific understanding of Ad has equipped researchers with unique engineering capabilities, resulting in successes in vector retargeting to defined cell types. 

Of particular note, two strategies have been developed to achieve Ad targeting to DCs, including the use of virus-cell adapter proteins and direct genetic modifications of the Ad vector [23]. These features in combination with the success of intranasal Ad as a vaccine have driven our interest in Ad as a platform for pandemic preparedness and therapeutic medicine. However, Ads possess limitations for diseases where rapid translation of the technology is required, especially in comparison to mRNA-based therapeutics. In the case of vaccines, a new Ad vector must be developed for each antigen, which involves cloning the antigen sequence into the Ad vector, transfection of DNA into a suitable cell line, development of seed virus stock, upscaling the vector in large bioreactors, and finally purification and release of the product. It would be highly advantageous to reduce this pipeline, especially for diseases such as cancer where the time-to-delivery for neoantigen cancer vaccines could dramatically impact a patient’s outcome.

These limitations have led us to explore combining mRNA with our Ad vector platform. We envision that an engineered Ad vector could be manufactured in bulk and then, used to deliver disease- or patient-specific mRNA, capitalizing on the targeting and intranasal delivery capacities of Ad while maintaining the molecular flexibility and ease of manufacture of mRNA therapeutics. We previously designed an Ad-polylysine (AdpL) system wherein plasmid DNA was packaged by polylysine via electrostatic association and attached to the exterior of the virion [24,25]. We recently expanded this strategy to mRNA and found that AdpL could achieve mRNA delivery both in vitro and in vivo [26]. Despite these successes, this approach exhibited a limited loading capacity for mRNA, and the chemical modifications to link the Ad and mRNA offered no control over the stoichiometry and location where the mRNA is linked to Ad surface. This may abrogate the Ads’ native tropism and further limit our ability to exploit this platform via rational surface engineering. In this study, we, therefore, developed an engineered Ad-mRNA platform, leveraging Catcher/Tag bioconjugates to attach mRNA to specific locations on the Ad capsid at the molecular level. 

## 2. Materials and Methods

### 2.1. Cells

A549 (ATCC CCL-185, Manassas, VA, USA) cells were grown in Dulbecco’s Modified Eagle Medium (DMEM) supplemented with 10% fetal bovine serum (FBS) and 1% 10,000 units/mL penicillin and 10,000 μg/mL streptomycin in a humidified incubator at 37 °C with 5% CO_2_ atmosphere.

### 2.2. Adenovirus 

The construction of Ad.hexon.SpyTag was described in our previous report [27]. Ad.hexon.SpyTag was engineered based on the chimpanzee adenovirus SAd36 with the early E1 region replaced by a CMV promoter-hybrid intron eGFP cassette and hexon hypervariable region five replaced by 48 bp SpyTag003, while the control virus did not have SpyTag incorporation. 

### 2.3. Proteins 

The SpyCatcher-SnoopCatcher fusion protein was derived from plasmid pET28a SpyCatcher-SnoopCatcher [28]. The pET28a SpyCatcher-SnoopCatcher was a gift from Mark Howarth (Addgene plasmid # 72324; http://n2t.net/addgene:72324 accessed on 1 October 2022; RRID: Addgene_72324). The recombinant plasmid from DH5α cells was isolated following the protocol in QIAprep Spin Miniprep Kit (QIAGEN) and then quantitated using NanoDrop Microvolume Spectrophotometers. *E. coli* BL21-CodonPlus (DE3)-RIPL competent cells (Thermo Fisher Scientific, St. Louis, MO, USA) were chosen for expressing the protein of interest. The purified plasmid was transformed into *E. coli* BL21-CodonPlus (DE3)-RIPL competent cells following the electroporation protocol. Single colonies were inoculated into LB broth containing the antibiotic required to maintain the expression plasmid and 50 µg/mL of chloramphenicol, specific for *E. coli* BL21-CodonPlus (DE3)-RIPL competent cells, for induction of protein expression. The next morning, a 25 mL culture was added into 500 mL of fresh LB broth without selecting antibiotics and incubated with shaking at 220–250 rpm at 37 °C for 2 h, and then IPTG was added to a final concentration of 1 mM. The mixture was incubated with shaking at 220–250 rpm at 37 °C for 2 h. The cells were spun down at 6000 rpm for 10 min at 4 °C, and the pellets were resuspended in 40 mL of PBS. Cell pellets were stored in −80 °C freezer for protein purification. The protein including the 6-his tag was purified using Ni-NTA Spin Columns (Thermo Fisher Scientific, St. Louis, MO, USA) and quantified by BCA Protein Quantification Kit (Thermo Fisher Scientific, St. Louis, MO, USA). 

SnoopTag linked to maltose binding protein (SnoopTag-MBP) was derived from pET28a SnoopTag-MBP and was produced following the same method as SnoopCatcher-SnoopCatcher [28]. The pET28a SnoopTag-MBP was a gift from Mark Howarth (Addgene plasmid # 72323; http://n2t.net/addgene:72323 accessed on 1 October 2022; RRID:Addgene_72323). SpyCatcher and SpyTag-MBP purified proteins were ordered from Kerafast (Boston, MA, USA). 

### 2.4. mRNA Binding Candidates

The truncated protamine and RALA peptides were synthesized as SnoopTag fusions by Wuxi AppTec (Wuhan, China) (SnoopTag-GS linker-protamine: GKLGDIEFIKVNKG-SGESGSG-RSQSRSRYYRQRQRSRRRRRRSR, SnoopTag-GS linker-RALA: GKLGDIEFIKVNKG-SGESGSG-WEARLARALARALARHLARALARALRACEA) dissolved in nuclease-free water to 3.1 mg/mL and aliquoted and stored at −80 °C. 

### 2.5. mRNA 

mCherry mRNA was purchased from TriLink Biotechnologies (San Diego, CA, USA), and mCherry circRNA was purchased from Creative Biogene (New York, NY, USA). Both were dissolved in nuclease-free water to 1.0 mg/mL and aliquoted and stored at −80 °C.

### 2.6. Catcher and Tag Reaction

The “AdPro” system was built upon the reaction between the catcher and tag. Each step was reacted at room temperature for 2 h, followed by loading 3× SDS-PAGE loading buffer and heating at 97 °C for 8 min. Then, the reaction process was revealed by performing 4−15% gradient SDS-PAGEs using the Criterion electrophoresis system, and gels were stained with coomassie blue dyes. 

### 2.7. RiboGreen Assay 

The RiboGreen assay was applied to quantify the protamine-mediated mRNA condensation level by Quant-it™ RiboGreen RNA Assay Kit (Thermo Fisher, St. Louis, MO, USA). The protamine-snoopTag/mRNA mixture was prepared at different ratios under room temperature by incubating for 2 h. Each sample was diluted in a microplate well to maintain the mRNA concentration within the dynamic range. The working solution was added to each microplate well and incubated for 2 min at room temperature and protected from light. The sample fluorescence was measured using a fluorescence microplate reader with the excitation wavelength at 480 nm and emission at 520 nm. The RNA standard curve was generated using the ribosomal RNA standard sample provided in the kit, and diffused mRNA concentration in different samples was calculated based on the standard line. mRNA condensation level was calculated by normalizing the protamine-mRNA samples to the naked mRNA samples. 

### 2.8. Ad-mRNA Constructs Preparation

To prepare the Ad-mRNA complexes, the indicated amount of Ad.hexon.SpyTag virus was incubated with SpyCatcher-SnoopCatcher at a molar ratio of 1:2, and protamine-snoopTag or RALA-Snooptag was incubated with mRNA at different ratios. The incubated reaction was performed at room temperature for 2 hrs. The two mixtures were then incubated together for another 2 h at room temperature. 

### 2.9. Gene Transfer Assay with A549 Cells 

A549 cells were plated in 96-well plates with a cell density of 2.5 × 10^−4^ cells per well 16 h before the treatment. To treat the cells, the culture medium was replaced with 2% FBS medium containing the Ad-mRNA formulations with various amounts of virus particles. Gene transfer analysis was performed 24 h post-infection.

Qualitative analysis was revealed by fluorescent images using an Olympus DP71 color microscope digital camera (Olympus, Shinjuku City, Tokyo, Japan). The optimal camera acquisition time for GFP and mCherry fluorescence was set using the untreated cells. Quantitative analysis was measured through an Attune NxT acoustic focusing cytometer (Thermo Fisher, USA). A549 cells were detached using Trypsin-EDTA and stained with Sytox Red for live/dead discrimination (Invitrogen, Waltham, MA, USA), and the untreated cells were used to set thresholds defining the background. 

### 2.10. Statistical Analyses

Specific methods used for data analysis are noted in the corresponding Figure legends. In all cases, analysis was carried out using GraphPad Prism 9 and the *t*-test to determine differences between two groups and one-way ANOVA to determine differences among multiple groups. A *p*-value of < 0.05 was used, and significance is indicated as “*”. A *p*-value of < 0.05 was used, and significance is indicated as *: *p* < 0.0322, **: *p* < 0.0021, ***: *p* < 0.0002, **** *p* < 0.0001, and ns: not significant. 

## 3. Results 

### 3.1. Design and Construction of the Engineered Ad-mRNA Binder Bioconjugate Complex

To achieve the binding of mRNA to specific locales on the Ad capsid, we leveraged “Catcher/Tag” protein bioconjugate technology. SpyTag is a 15-amino acid peptide that forms a spontaneous covalent bond with its 110-amino acid long protein partner SpyCatcher [28,29,30]. The reaction between Tag and Catcher occurs under broad conditions upon simple mixing and exhibits a very high reaction yield, resulting in an advantageous system for protein engineering. We previously developed Ads that genetically incorporate SpyTag into the hexon protein, which is the major capsid structural component with 720 copies forming the virus particle. We utilized this vector to successfully deliver an engineered recombinant protein, SpyCatcher-Cas9, via anchoring to hexon [27]. Herein, we, therefore, employed this SpyTag-modified Ad as an anchoring platform to attach mRNA binding proteins to the Ad capsid, enabling subsequent site-specific attachment of mRNA to the virus exterior. 

In this regard, cell-penetrating peptides (CPPs) have been identified as effective mRNA condensing reagents, e.g., polylysine, protamine, and RALA [31,32,33]. To anchor these mRNA binding candidates to the hexon locale of the Ad capsid, we first considered the development of SpyCatcher-CPP recombinant fusion proteins. However, due to the fact that most CPPs are positively charged peptides less than 20 amino acids in length and rich in arginine or lysine, directly manufacturing SpyCatcher-CPP recombinant proteins in E. Coli proved challenging in our preliminary studies. In addition, assessing the mRNA binding ability of each CPP candidate via SpyCather-CPP requires de novo protein production, including plasmid design, an appropriate expression system for each CPP, and isolation and quantification of the desired peptides. In this case, screening a wide selection of CPP candidates is relatively time-consuming and labor-intensive. 

**Figure 1:**
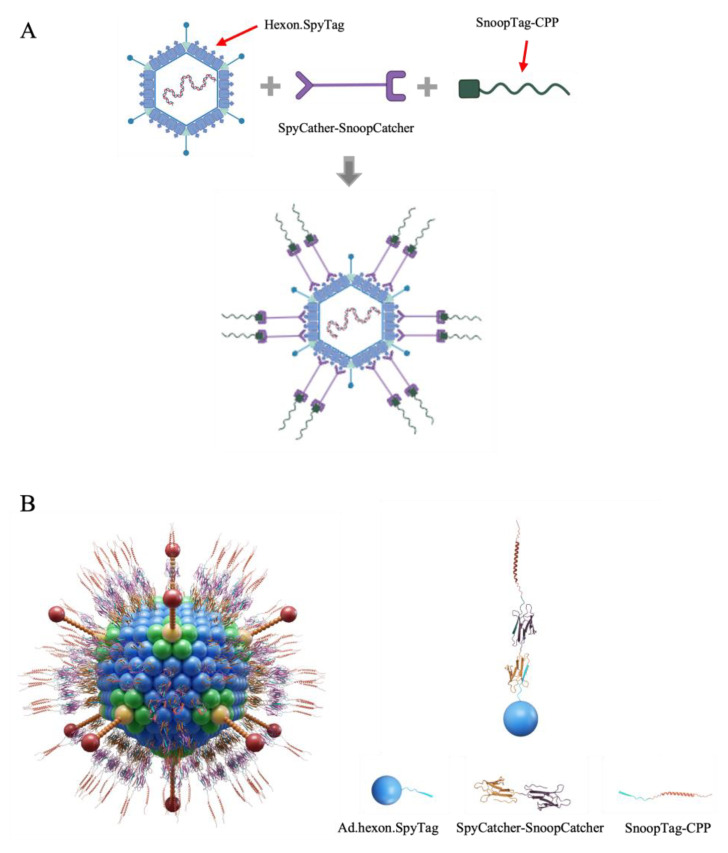
Ad-piggyback mRNA binder. (**A**) Schematic illustration of the construction process of Ad-piggyback mRNA binder via Catcher/Tag molecular glue. (**B**) Model of Ad-piggyback mRNA binder system generated by 3ds Max software 2022.

Considering all the factors above, we introduced an intermediate linker, SpyCatcher-SnoopCatcher, to circumvent this problem. SnoopCatcher forms a spontaneous isopeptide bond with its partner SnoopTag but has no cross-reaction with SpyCatcher/SpyTag [28]. By virtue of this, the SpyCatcher-SnoopCatcher fusion can specifically link to SpyTag-modified Ad at one end, and any molecule including a SnoopTag at the other. By using the SnoopTag-CPP adapter peptides we were able to avoid having to produce SpyCatcher-CPP recombinant proteins. Notably, SnoopTag-CPPs are approximately 40 amino acids in length which can be achieved by short peptide synthesis technology, allowing us to directly obtain various SnoopTag-CPPs in a rapid and accurate manner and consequently speed the process of screening candidate mRNA binders. The Ad-mRNA binder bioconjugate platform is thus designed to consist of SpyTag-modified Ad, the SpyCatcher-SnoopCatcher intermediate linker, and a SnoopTag-fused CPP (Figure 1). 

**Figure 2:**
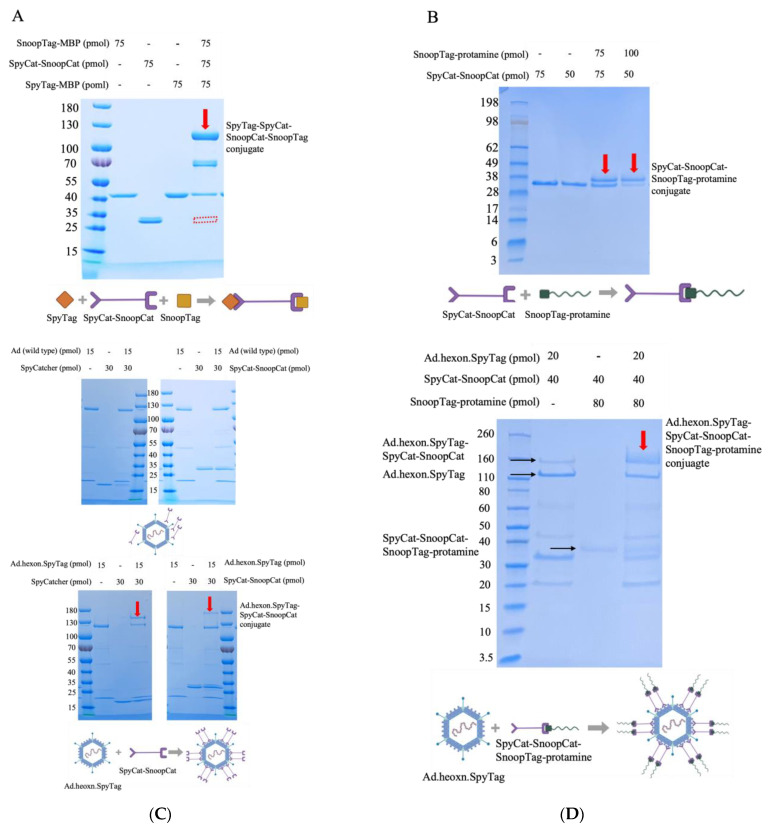
Construction process of the “Ad-piggyback protamine” complex. (**A**) Quality evaluation of SpyCat-SnoopCat intermediate linker. SnoopTag-MBP, SpyCat-SnoopCat, and SpyTag-MBP were incubated at room temperature for 2 h with a molar ratio of 1:1:1. (**B**) Formation of SpyCat-SnoopCat-SnoopTag-protamine conjugate. SnoopTag-protamine fused peptide was incubated with SpyCat-SnoopCat at room temperature for 2 h with a molar ratio of 1:1 and 2:1. (**C**) Anchoring of SpyCat-SnoopCat to hexon locales of Ad capsid and the formation of Ad.hexon.SpyTag-SpyCat-SnoopCat complex. Top: SpyCat or SpyCat-SnoopCat was incubated with wild-type Ad (non-SpyTag integrated) at room temperature for 2 h with a molar ratio of 2:1. Bottom: SpyCat or SpyCat-SnoopCat was incubated with Ad.hexon.SpyTag (SpyTag-integrated) at room temperature for 2 h with a molar ratio of 2:1. (**D**) Formation of “AdPro” or Ad.hexon.SpyTag-SpyCat-SnoopCat-SnoopTag-protamine complex. Ad.hexon.SpyTag, SpyCat-SnoopCat, and SnoopTag-protamine were incubated at room temperature for 2 h with a molar ratio of 1:2:4.

We selected protamine as an mRNA binding protein, as protamine has been demonstrated to serve as an effective mRNA condensing agent for delivery to various cell types and has been used in clinical trials [34,35,36,37,38]. To construct the “Ad-mRNA binder” complex, we first examined the quality of the intermediate linker, SpyCatcher-SnoopCatcher (SpyCat-SnoopCat hereafter) via gel shift assays. SpyCat-SnoopCat was incubated with SpyTag-MBP and SnoopTag-MBP for 2 h, which resulted in a complete reaction as evidenced by the band shifting from the original site to the desired site (indicated in red), with the molecular weight corresponding to the SpyTag-MBP-SpyCat-SnoopCat-SnoopTag-MBP protein conjugate (Figure 2A). Therefore, the SpyCat-SnoopCat fusion provides a robust system for specific and irreversible linkage of proteins containing SpyTag and SnoopTag. 

We next assessed the binding efficiency of the fusion with SnoopTag-fused protamine by incubating SpyCat-SnoopCat with SnoopTag-protamine at two different ratios. We found that increasing the amount of SnoopTag-protamine enhanced the reaction efficiency. As an initial step towards actualizing the “Ad-mRNA binder” bioconjugate, the SpyCat-SnoopCat-SnoopTag-protamine conjugate was successfully constructed (Figure 2B). 

We next evaluated the binding efficiency of SpyCat-SnoopCat to the Ad.hexon.SpyTag virus. SpyCatcher and SpyCat-SnoopCat were incubated separately with Ad.hexon.SpyTag at a ratio of 1:2, and we observed that both proteins efficiently bound to the hexon, although SpyCat-SnoopCat exhibited a relatively lower reactivity (Figure 2C, bottom). In contrast, the wild-type Ad (non-SpyTag integrated), used as the control, had no interaction with either SpyCatcher or SpyCat-SnoopCat (Figure 2C, top). Having validated the binding specificity and efficiency of SpyCat-SnoopCat to Ad.hexon.SpyTag and to SnoopTag-protamine, we next constructed the “Ad-mRNA binder” bioconjugate. A mixture of Ad.hexon.SpyTag, SpyCat-SnoopCat, and SnoopTag-protamine were incubated at a molar ratio of 1:2:4. Migration of the conjugate hexon.SpyTag-SpyCat-SnoopCat-SnoopTag-protamine band to the desired site (indicated in red) demonstrated the successful construction of Ad.hexon.SpyTag-SpyCat-SnoopCat-SnoopTag-protamine (AdPro) complex (Figure 2D). 

### 3.2. “Ad-Bioconjugate” System-Mediated mRNA Delivery In Vitro 

**Figure 3:**
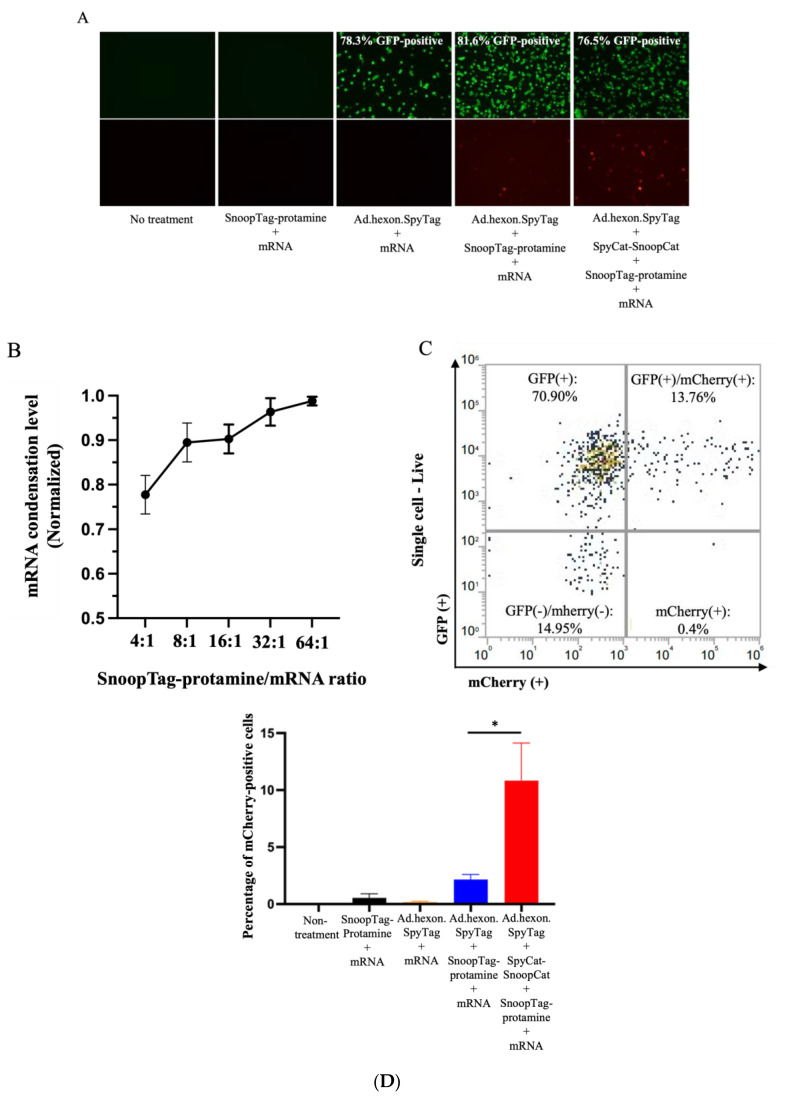
Ad-piggyback protamine mediated effective mRNA delivery in vitro. Engineered Ad with SpyTag incorporated into the hexon locales on the capsid protein serves as an anchoring platform for the protamine/mRNA complex to attach. Ad encodes the GFP gene, and the piggybacked mRNA encodes mCherry fluorescent protein gene. The contribution of each component to effective mRNA delivery is examined by setting the following groups: Group 1: non-treatment; Group 2: mRNA 0.5 μg, SnoopTag-protamine/mRNA = 8:1(mass ratio); Group 3: Ad.hexon.SpyTag 2 × 10^−8^ vp, mRNA 0.5 μg; Group 4: Ad.hexon.SpyTag 2 × 10^−8^ vp, mRNA 0.5 μg, SnoopTag-protamine/mRNA = 8:1 (mass ratio); Group 5: Ad.hexon.SpyTag 2 × 10^−8^ vp, SpyCat-SnoopCat/Ad.hexon.SpyTag = 2:1 in molar ratio, mRNA 0.5 μg, SpyTag-protamine/mRNA = 8:1 (mass ratio). * *p* ≤ 0.05. (**A**) Qualitative analysis of gene transfer as visualized by fluorescent images. (**B**) Assessment of mRNA condensation level by RiboGreen assay. (**C**) Representative flow cytometry density plot from a sample in Group 5. (**D**) Quantitative analysis of mRNA delivery efficiency by flow cytometry. Indicated groups were analyzed using a standard *t*-test.

We next assessed the ability of the “AdPro” system to deliver mRNA in vitro. We employed Ad.hexon.SpyTag encoding a GFP reporter gene and mRNA encoding mCherry fluorescent protein, allowing us to independently analyze both Ad-mediated and mRNA-mediated gene delivery. Considering that several components existed in the Ad-mRNA bioconjugate system, we attempted to explore the contribution of each component to effective mRNA delivery. To this end, five groups were evaluated, including no treatment (control), SnoopTag-protamine/mRNA, Ad.hexon.SpyTag/mRNA, Ad.hexon.SpyTag/SnoopTag-protamine/mRNA, and Ad.hexon.SpyTag -SpyCat- SnoopCat-SnoopTag-protamine/mRNA (“AdPro”). Based on analysis of fluorescent images, bioconjugation of mRNA on the Ad surface did not abrogate Ad-mediated gene transfer. Furthermore, the “AdPro” system exhibited promising mRNA delivery ability as indicated by the presence of mCherry-positive cells (Figure 3A).

Having qualitatively confirmed the capacity of our system to achieve mRNA delivery, we next analyzed the mRNA delivery efficiency via quantitative flow cytometry. We first hypothesized that the mRNA condensation level would be a critical determinant of the overall delivery efficiency. We thus varied the ratio of protamine-SnoopTag and mRNA to confirm effective condensation. We assessed condensation via a RiboGreen assay, hypothesizing that condensed mRNA would be unable to bind with the reagent and would show reduced fluorescence compared to unprotected mRNA, allowing us to quantify the protamine-mediated mRNA condensation level. Compared to the naked mRNA group, groups involving protamine-SnoopTag showed reduced mRNA amounts, indicating that SnoopTag-fused protamine could potently condense mRNA and block binding of the RiboGreen reagent. Additionally, we found that increasing the ratio of protamine-SnoopTag/mRNA increased the mRNA condensation level, as expected, with a mass ratio of 8:1 presenting approximately 90% condensation ability with relatively low protamine consumption (Figure 3B). We thus applied this ratio to experimental groups involving protamine-SnoopTag and mRNA and used flow cytometry to identify the differences in mRNA delivery efficiency among these groups. The complete “AdPro” system presented higher mRNA delivery efficiency than the other formulations. The SnoopTag-protamine/mRNA group had negligible mRNA delivery, which underscores the functional role of Ad in this system. Additionally, although Ad.hexon.SpyTag/SnoopTag-protamine/mRNA also exhibited a certain degree of mRNA delivery ability, the mRNA delivery efficiency was significantly lower than that of the “AdPro” system. (Figure 3C,D). 

### 3.3. “AdPro” System-Mediated circRNA Delivery In Vitro 

**Figure 4:**
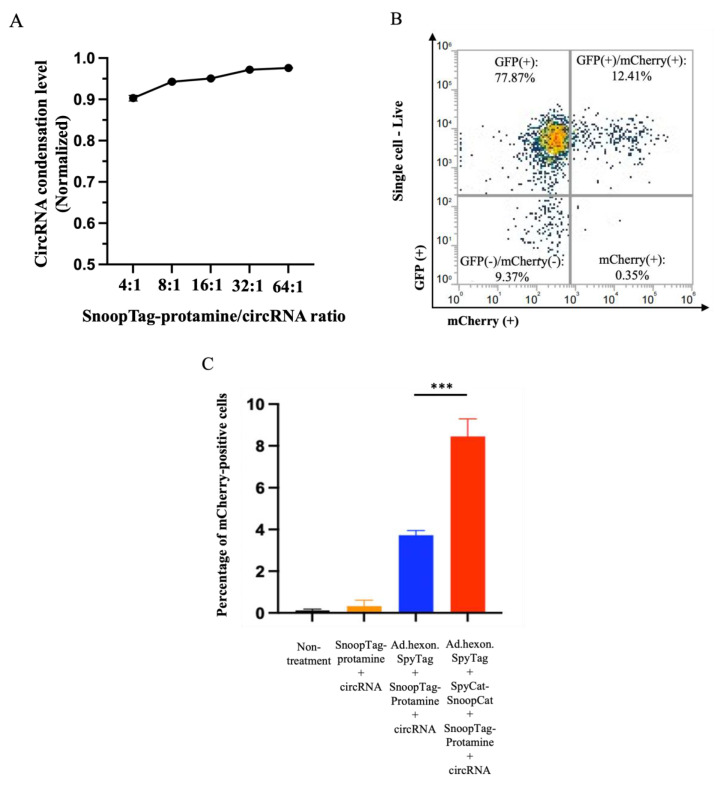
“AdPro” system mediated circRNA delivery in vitro. Engineered Ad with SpyTag incorporated into the hexon locales on the capsid protein serves as an anchoring platform for the protamine/circRNA complex to attach. Ad encodes the GFP gene, and the piggybacked circRNA encodes mCherry fluorescent protein gene. The contribution of each component to effective circRNA delivery is examined by setting the following groups: Group 1: Non-treatment; Group 2: circRNA 0.5 μg, SnoopTag-protamine/circRNA 8:1(mass ratio); Group 3: Ad.hexon.SpyTag 2 × 10^−8^ vp, circ RNA 0.5 μg, SnoopTag-protamine/mRNA = 8:1(mass ratio); Group 4: Ad.hexon.SpyTag 2 × 10^−8^ vp, SpyCat-SnoopCat/Ad.hexon.SpyTag = 2:1 (in molar ratio), circ RNA 0.5 μg, SpyTag-protamine/mRNA = 8:1(mass ratio). (**A**) Assessment of circRNA condensation level by RiboGreen assay. (**B**) Representative flow cytometry density plot from a sample in group 4. (**C**) Quantitative analysis of circRNA delivery efficiency by flow cytometry. Indicated groups were analyzed using a standard *t*-test.

Having demonstrated that the AdPro system could achieve gene transfer of linear mRNA, we also attempted to deliver an alternative RNA vector species, circular RNA (circRNA). We again performed a condensation assay using RiboGreen and found that protamine was able to offer similar condensation levels with circRNA as linear mRNA (Figure 4A). Similarly, we studied the contribution of each component of the AdPro system to circRNA delivery and found that the complete AdPro system showed the highest delivery efficiency, as expected (Figure 4B,C). These studies demonstrate the cargo flexibility of the AdPro system to deliver multiple nucleic acid types. 

### 3.4. Strategies to Optimize Intracellular mRNA Release

**Figure 5:**
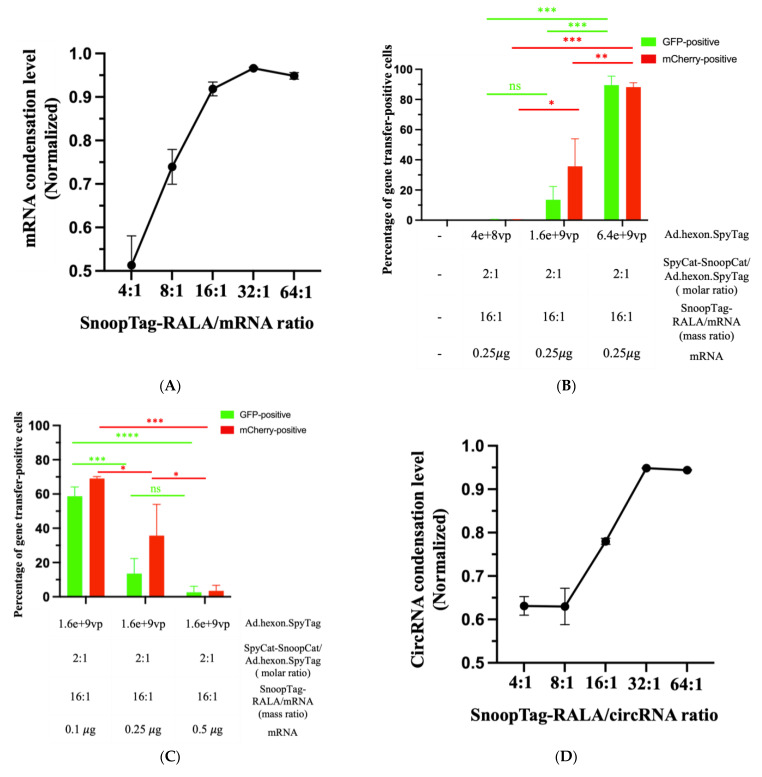

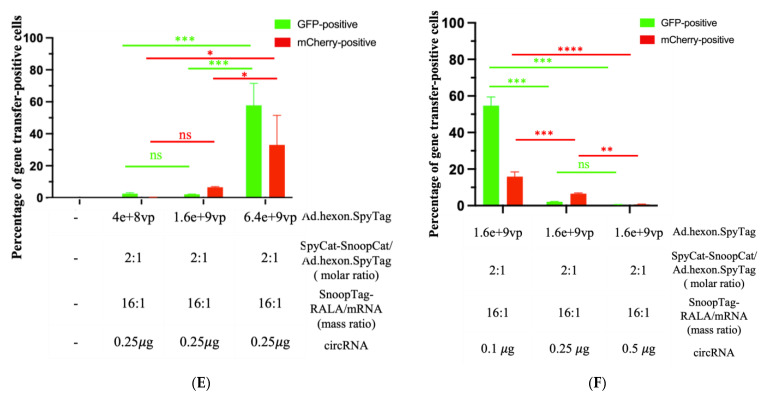
The “AdRALA” system achieved higher gene transfer. (**A**) Assessment of mRNA condensation level by RiboGreen assay. (**B**,**C**) Quantitative analysis of gene transfer efficiency by flow cytometry. (**D**) Assessment of circRNA condensation level by RiboGreen assay. (**E**,**F**) Quantitative analysis of gene transfer efficiency by flow cytometry. Statistical comparisons were obtained via one-way ANOVA with Tukey’s multiple comparisons test.

RALA is an advanced version of protamine with a unique sequence and structural organization demonstrating enhanced cytosol translocation of mRNA by virtue of the RALA motif compared to other arginine-rich peptides. The RALA motif has been found to bind mRNA with appropriate strength such that mRNA is well condensed and protected but ribosomal binding upon cytosolic delivery is not compromised. These are the two pivotal factors that impact the mRNA translation efficacy [7]. We therefore attempted to optimize the Ad-mRNA system by simply synthesizing a RALA-SnoopTag peptide and thereby effectively substituting protamine with RALA. We performed a RiboGreen condensation assay as before to identify the mRNA condensing ability of RALA. Various ratios of SnoopTag-fused RALA/mRNA were assessed, and we found that increasing the ratio led to higher mRNA condensation levels, as expected, and a ratio of 16:1 was able to offer a potent condensation of approximately 92% with a relatively low amount of RALA applied (Figure 5A). According to this, a SnoopTag-RALA/mRNA ratio of 16:1 was employed in further studies. 

Recognizing that protamine-condensed mRNA was piggybacked to the Ad’s surface, we hypothesized that mRNA delivery might take advantage of the cell-entry mechanism of Ad [39]. Intrigued by this, we applied various Ad doses to a fixed mCherry mRNA amount at 0.25 μg and analyzed Ad and mRNA gene transfer. As might be expected, we found that increasing the amount of Ads enhanced Ad-mediated GFP expression. In company with increased Ad-mediated gene transfer, mRNA-mediated mCherry expression was notably enhanced. Furthermore, the “AdRALA” platform presented a potent mRNA delivery capacity, achieving approximately 90% efficiency in both Ad-mediated gene transfer and mRNA gene transfer (Figure 5B). 

We next hypothesized that another factor determining the gene transfer efficiency of our system might be the structure of the “AdRALA” complex. Specifically, if the piggybacked mRNA were overloaded on the Ad surface, the Ad-mediated cell entry and endosome escape could be compromised due to a lack of association between the Ad capsid proteins and cell/endosome membranes, which would subsequently impact both Ad-mediated and mRNA gene transfer [24,25,39]. Based on this assumption, we fixed the amount of virus (16 × 10^−8^ vp) that achieved moderate Ad-mediated gene transfer in Figure 5B and manipulated the mRNA doses at 0.1 µg, 0.25 µg, and 0.5 µg. We found that compared with the group treated with 0.25 µg mRNA both Ad and mRNA gene transfers were reduced to approximately 3% when 0.5 µg mRNA was used to formulate the Ad-mRNA bioconjugate. In contrast, the group with 0.1 µg mRNA applied surprisingly showed the highest efficiency among the groups with approximately 59% Ad-mediated gene transfer and 69% mRNA delivery (Figure 5C). 

Having established success using AdRALA to deliver linear RNA, we also attempted circRNA delivery. We again used the RiboGreen assay to evaluate the circRNA condensation levels by SnoopTag-fused RALA and selected a RALA/circRNA ratio of 16:1 (Figure 5D). Having established success using AdRALA to deliver linear RNA, we also attempted circRNA delivery. We again used the RiboGreen assay to evaluate the circRNA condensation levels by SnoopTag-fused RALA and selected a RALA/circRNA ratio of 16:1 (Figure 5D). We employed the same experimental settings as linear RNA in the context of circRNA and achieved 58% Ad-mediated and 33% circRNA gene transfer by modulating the mRNA and virus ratios as before (Figure 5E,F).

## 4. Discussion

In this study, we designed an adenovirus-based platform that utilizes Catcher/Tag molecular glue to accomplish the site-specific linkage of mRNA to the Ad capsid at highly precise locales. We characterized the construction process of the “Ad-mRNA” bioconjugate in a bottoms-up approach based on the irreversible bond formation between Catcher and Tag and demonstrated the necessary contribution of each component to successful mRNA delivery. We showed the feasibility of employing the “Ad-mRNA” system to deliver both standard linear mRNA and circRNA. Furthermore, we optimized the system by improving several conditions, including replacing protamine with an advanced mRNA binder RALA, identifying the optimal mRNA condensation levels, and fully exploiting the Ad-mediated cell entry and endosome escape ability via varying applied doses of Ad and mRNA. 

In our AdPro design, the protamine/mRNA complex was piggybacked to Ad surface via the intermediate linker SpyCat-SnoopCat forming covalent bonds between Tag engineered into virus and protamine. We noticed that the formulation without the linker still exhibited certain degrees of linear mRNA and circRNA delivery. However, the complete AdPro formulation had higher delivery efficiency (Figure 3D and Figure 4C). This highlights the rational design of the AdPro system, wherein the covalent linkage of the virus and mRNA contributed by SpyCat-SnoopCat plays an essential role. This may be attributed to the fact that the covalent linkage stabilizes the Ad-mRNA construct and, therefore, makes it more resistant to the exterior environment during intracellular trafficking, allowing for exploiting Ad function to augment the complex internalization and gene transfer. 

Furthermore, our system was highly flexible in its ability to deliver different types of nucleic acids. In both protamine and RALA contexts, both peptides offered similar condensation levels for linear mRNA and circRNA. However, the delivery efficiency of circRNA was relatively lower than that of linear mRNA even with the same experimental settings. We hypothesize that the structural differences between linear and circular RNA may impact the cell entry and translation processes. To investigate the mechanism behind this, in-depth studies may include advanced imaging technology to visualize the process of complex attachment to the cell surface and intracellular trafficking. 

In our system, the mRNA binding peptide may be a critical opportunity for optimization due to its central role in condensing the mRNA, attaching it to the virus capsid, and then releasing mRNA in the cytosol. Promising mRNA binding binders should efficiently condense mRNA but retain the accessibility of ribosomal binding upon cytosolic delivery of mRNA. RALA was found to bind mRNA with such strength that it leverages these two functions [33]. We thus employed RALA in our delivery system. Other groups have exploited rational peptide design to understand the essential functional amino acids in RALA via residue replacement, seeking improvement to the RALA peptide. Further studies may consider evaluating these RALA-analogs in our system [39]. In addition, the Ad capsid proteins play pivotal functional responsibilities in mediating cell entry and endosome disruption to translocate the mRNA cargo to the cytosol [40]. We leveraged the Ad functional platform in these two aspects while accomplishing potent mRNA package capacity by increasing Ad doses and identifying appropriate mRNA loading doses, allowing us to achieve high efficiency in both Ad-mediated gene transfer and mRNA gene transfer. While we have achieved potent mRNA delivery after several optimization steps, aspects of the technology must be improved in future work. For example, the excess number of SpyCatcher-SnoopCatcher molecules added during the complex preparation could potentially lead to aggregation issues. Purifying Ads after conjugation could mitigate this problem and is also a key step for improving the complex purity and demonstrating the clinical feasibility.

As manifested in our experiments, a key advantage of the “Ad-mRNA” system is its modular design, embodying high flexibility and compatibility in replacing each module, including Ad vectors and locations of the Tag, the SpyCat-SnoopCat linker, mRNA binders, and genetic materials. Building on this point, we were able to easily switch the genetic material delivered from linear mRNA to circRNA and evaluated different mRNA binders involving protamine and RALA. This concept opens the door to advancing the Ad-mRNA system further by modifying each component of the system. In this study, we employed Ad with SpyTag integrated into the hexon, but our group and others have characterized Ads with SpyTag and another peptide, DogTag, on multiple other capsid proteins, which could be employed in the AdPro system with a matching intermediate linker [41]. Broadly speaking, this design could be applied to any viral vector or virus-like particles that include exposed Tag peptides.

The innovative design of incorporating “Catcher/Tag” molecular glue technology in the AdPro system allows for manipulating, characterizing, and accomplishing site-specific binding of mRNA on Ad capsid locales and opens the opportunity for engineering other Ad capsid proteins, enabling us to fully exploit the Ad platform. Decades of research with Ads have resulted in a multitude of studies describing the redirection of virus tropism towards desirable cell types via fiber modifications. To this end, future work may include engineering Ads to achieve cell-targeted mRNA delivery. Specifically, our group has characterized Ads with DC-targeting moieties on the fiber and developed DC-targeted vaccines based on this [23]. Building upon our previous work, utilizing Ads that incorporate SpyTag modification on the hexon serving as an mRNA package platform and DC-targeting moieties on the fiber to achieve DC-targeted mRNA delivery seems to be within reach. In addition, given that Ads have been successfully used as intranasal vaccines in the clinic, we could exploit this capacity to achieve intranasal vaccination with mRNA. Some Ads species, which possess natural abilities to infect cells in the upper respiratory tract, hold promise for trafficking mRNA across the mucosal barrier and achieving gene expression when combined with our delivery system. 

## Data Availability

The data presented in this study are available on request from the corresponding author.
